# 06. United States Healthcare Provider Preferences for Adult Pneumococcal Vaccine Recommendations

**DOI:** 10.1093/ofid/ofab466.209

**Published:** 2021-12-04

**Authors:** Jeffrey Vietri, Kelley Meyers, Christine Poulos, Erica Chilson, Carolyn Sweeney, Kimberly Davis, Vincenza Snow

**Affiliations:** 1 Pfizer Inc, Collegeville, Pennsylvania; 2 RTI-Health Solutions, Research Triangle Park, NC; 3 Pfizer, 500 Arcola Road, Pennsylvania; 4 RTI-HS, Research Triangle Park, North Carolina; 5 Pfizer Vaccines, Collegeville, PA

## Abstract

**Background:**

Pneumococcal vaccine recommendations for US adults are complex, varying by age and underlying conditions, and include both 23-valent polysaccharide vaccine (PPSV23) and 13-valent pneumococcal conjugate vaccine. The Advisory Committee on Immunization Practices (ACIP) will vote on new recommendations in October after the 15- (PCV15) and 20-valent (PCV20) conjugate vaccines are approved. Stakeholder acceptability is part of ACIP’s evidence to recommendation framework, but few data are available on health care providers’ (HCPs) preferences for potential recommendations.

**Methods:**

752 HCPs (300 physicians, 150 nurse practitioners, 150 physician assistants, & 152 pharmacists) were surveyed. Object case best-worst scaling (BWS) was used to elicit preferences for hypothetical recommendations for 1) adults 19-64 years with chronic conditions and 2) immunocompetent adults ≥65 years. Presented recommendations included combinations of PCV15/PCV20 either as routine or after shared clinical decision making (SCDM), and PPSV23 as routine, SCDM, or no recommendation. Following BWS, HCPs were asked to assume ACIP was considering implementing both of their preferred recommendations for the age/risk groups. HCPs were then given the opportunity to change their selections and propose recommendations not included in the BWS exercise. Additional information was collected using conventional survey items.

**Results:**

Routine use of higher-valent PCVs in sequence with PPSV23 was most often preferred for both adults 19-64 with chronic conditions (40%) and immunocompetent adults ≥65 (49%) when elicited separately for each age/risk group. Most respondents (63%) revised their recommendations after considering implementation, which resulted in most (59%) favoring recommendations harmonized across the age/risk groups, and 75% favoring routine use of PCV15 or PCV20 among immunocompetent adults ≥65. When asked directly, HCPs generally approved of the idea of simplifying adult pneumococcal vaccine recommendations, harmonizing the interval between vaccines, and lowering the cutoff for age-based recommendations below 65 years.

**Conclusion:**

US HCPs generally prefer simplification of the adult pneumococcal recommendation, favoring broad routine use of both higher-valent PCVs and PPSV23.

**Disclosures:**

**Jeffrey Vietri, PhD**, **Pfizer Inc** (Employee, Shareholder) **Kelley Meyers, PhD**, **RTI Health Solutions** (Independent Contractor) **Christine Poulos, PhD**, **Pfizer Inc** (Other Financial or Material Support, Employee of RTI-HS, which received funds from Pfizer to conduct the study) **Erica Chilson, PharmD**, **Pfizer, Inc** (Employee, Shareholder) **Vincenza Snow, MD**, **Pfizer Vaccines** (Employee)

